# Healthcare provider knowledge, attitudes, beliefs, and practices surrounding the prescription of opioids for chronic non-cancer pain in North America: protocol for a mixed-method systematic review

**DOI:** 10.1186/s13643-018-0858-7

**Published:** 2018-11-13

**Authors:** Joshua A. Rash, Norman Buckley, Jason W. Busse, Tavis S. Campbell, Kim Corace, Lynn Cooper, David Flusk, Alfonso Iorio, Kim L. Lavoie, Patricia A. Poulin, B. Skidmore

**Affiliations:** 1 Department of Psychology, Memorial University of Newfoundland, 230 Elizabeth Ave, St. John’s, NL A1B 3X9 Canada; 2 Department of Anesthesia, McMaster University, Hamilton, ON Canada; 3 Department of Health Research Methods, Evidence and Impact, McMaster University, Hamilton, ON Canada; 4 The Michael G. DeGroote Institute for Pain Research and Care, McMaster University, Hamilton, ON Canada; 5 The Michael G. DeGroote Centre for Medicinal Cannabis Research, McMaster University, Hamilton, ON Canada; 6 Department of Psychology, University of Calgary, Calgary, AB Canada; 7 The Royal Ottawa Mental Health Centre, Ottawa, ON Canada; 8 Department of Psychiatry, University of Ottawa, Ottawa, ON Canada; 9 University of Ottawa Institute of Mental Health Research, Ottawa, ON Canada; 10 Canadian Injured Workers Alliance, Thunder Bay, ON Canada; 11 Department of Anesthesia, Memorial University of Newfoundland, St. John’s, NL Canada; 12 Department of Psychology, University of Quebec at Montreal, Montreal, QC Canada; 13 Montreal Behavioral Medicine Centre (MBMC), Centre intégrée universitaire de santé et services sociaux de Nord de l’Ile de Montreal (CIUSSS-NIM), Hopital du Sacre-Coeur de Montreal, Montreal, QC Canada; 14 The Ottawa Hospital Research Institute, Ottawa, ON Canada; 15 The Ottawa Hospital Pain Clinic, Ottawa, ON Canada; 16 School of Psychology and Department of Anesthesiology and Pain Medicine, University of Ottawa, Ottawa, ON Canada; 17 Independent Information Specialist, Ottawa, ON Canada

**Keywords:** Clinical inertia, Clinical practice guideline adherence, Opioids, Chronic pain, Systematic review

## Abstract

**Background:**

Evidence from diverse areas of medicine (e.g., cardiovascular disease, diabetes) indicates that healthcare providers (HCPs) often do not adhere to clinical practice guidelines (CPGs) despite a clear indication to implement recommendations—a phenomenon commonly termed clinical inertia. There are a variety of reasons for clinical inertia, but HCP-related factors (e.g., knowledge, motivation, agreement with guidelines) are the most salient and amenable to intervention aimed to improve adherence. CPGs have been developed to support the safe and effective prescription of opioid medication for the management of chronic non-cancer pain. The extent of physician uptake and adherence to such guidelines is not yet well understood. The purpose of this review is to synthesize the published evidence about knowledge, attitudes, beliefs, and practices that HCPs hold regarding the prescription of opioids for chronic non-cancer pain.

**Methods:**

An experienced information specialist will perform searches of CINAHL, Embase, MEDLINE, and PsycINFO bibliographic databases. The Cochrane library, PROSPERO, and the Joanna Briggs Institute will be searched for systematic reviews. Searches will be performed from inception to the present. Quantitative and qualitative study designs that report on HCP knowledge, attitudes, beliefs, or practices in North America will be eligible for inclusion. Studies reporting on interventions to improve HCP adherence to opioid prescribing CPGs will also be eligible for inclusion. Two trained graduate-level research assistants will independently screen articles for inclusion, perform data extraction, and perform risk of bias and quality assessment using recommended tools. Confidence in qualitative evidence will be evaluated using the Grades of Recommendation, Assessment, Development, and Evaluation-Confidence in the Evidence from Qualitative Reviews (GRADE-CERQual) approach. Confidence in quantitative evidence will be assessed using the GRADE approach.

**Discussion:**

The ultimate goal of this work is to support interventions aiming to optimize opioid prescribing practices in order to prevent opioid-related morbidity and mortality without restricting a HCP’s ability to select the most appropriate treatment for an individual patient.

**Systematic review registration:**

PROSPERO CRD42018091640.

**Electronic supplementary material:**

The online version of this article (10.1186/s13643-018-0858-7) contains supplementary material, which is available to authorized users.

## Background

The prescription of opioid analgesics represents a double-edged sword. On the one hand, opioid analgesics (e.g., morphine, oxycodone, fentanyl) have been shown to provide modest improvement in pain and function for patients with chronic non-cancer pain. On the other hand, prescription opioids may lead to opioid-induced algesia [1, 2], addiction, or diversion, particularly at high doses (e.g., ≥ 200 mg morphine equivalents/day) [3]. As such, prescription opioids are associated with serious and increasing public health problems, such as addiction treatment admissions and overdose death [4].

### Opioid prescribing within the context of chronic pain management

In 2011, the United States Institute of Medicine concluded that chronic pain, defined as pain that persists longer than 3 months, or beyond the expected duration of healing [5], is a public health concern and should be treated as a disease itself [6]. While estimates vary depending on survey methodology, nationally representative data from Canada, the USA, Germany, and other European countries indicates that 20 to 30% of adults (≥ 18 years of age) suffer with chronic non-cancer pain [7–11]. The prevalence of chronic pain is higher among women [12] and ethnic minorities [13] and increases with age. Approximately 65% of community-dwelling seniors and 80% of older adults living in care facilities experience chronic pain, including cancer-related pain [14].

The goal of pain management is to decrease pain and improve function while monitoring for adverse effects [15]. In the late 1980s and early 1990s, the under-treatment of pain, including among patients with chronic non-cancer pain, garnered national attention in both the USA and Canada. A classic 1986 publication describing the treatment of chronic pain in 38 patients concluded that opioid pain relievers could be prescribed safely on a long-term basis [16]. Further, a letter to the editor of the New England Journal of Medicine asserted that the rate of addiction in patients receiving opioids was low [17]. This letter was heavily and uncritically cited as evidence that addiction was rare with long-term opioid therapy, many citing rates ≤ 1% [18]. Despite low-quality evidence, these sources were widely cited to support the expanded use of opioid medication for the management of chronic pain [4]. By the late 1990s, healthcare providers (HCPs) were encouraged by pharmaceutical companies and medical boards to be more proactive in treating all types of pain (e.g., acute, palliative, chronic) to alleviate suffering, including the prescription of opioid analgesics at relatively high doses for long durations [19]. The potential adverse effects of long-term opioid use were under-appreciated and often based on beliefs that opioids were safe for extended use in patients with chronic pain with no known dosing threshold. Opioid prescribing increased in a marked linear fashion until 2013 when it began to plateau in the USA [20] and Canada [21].

### Trends in opioid prescribing morbidity and mortality

The past two decades have been characterized by a linear increase in the prescription of opioid medications. A twofold increase in the consumption of hydrocodone and fivefold increase in the use of oxycodone was observed in the USA between 1999 and 2011 [22]. Observational data show a mean increase from 180 mg morphine equivalents per person in the US population per year in 1997 to 710 mg per person per year in 2010 [23]. This corresponded with a fourfold increase in the sale of prescription opioids [23], a fivefold increase in drug treatment admissions for prescription opioids (from ~ 20,000 to ~ 120,000) [24], more than a twofold increase in emergency department visits related to pharmaceutical opioids [25], and a fourfold increase in opioid-related overdose [26]. Although there is an undeniable rise in the availability of illicit opioids [27, 28], it has also been argued that opioid-related mortality is directly associated with the increase in opioid prescriptions observed in Canada, the USA, Europe, the UK, Spain, France, and Australia [21, 29–31]. Beyond the direct association, prescriber adjustment and tapering of opioid analgesics have been associated with risk of nonmedical use, morbidity, and mortality as individuals turn to illicit opioids in an attempt to manage their pain [32].

A systematic review of data from the USA and Canada identified three interacting factors associated with opioid-related mortality: (1) prescriber behaviors, (2) patient characteristics, and (3) systemic determinants [33]. Pertinent for this protocol, four ways that prescriber behaviors influence opioid-related mortality were elucidated. First, results from seven studies indicated that prescribing higher doses of opioids is associated with opioid-related mortality. Second, seven studies reported an association between more potent opioids, such as fentanyl, and opioid-related mortality. Third, 14 studies reported that the co-prescription of opioids with sedatives or more than one opioid was associated with the observed increase in opioid-related mortality. This was particularly relevant with the co-prescription of methadone which has a small window between therapeutic and fatal doses. Finally, eight studies reported that an increase in the number of opioid prescriptions played a role in opioid mortality through increased availability. The top quintile of prescribers was observed to issue opioid prescriptions 4.5 times more frequently than the next quintile and wrote the final prescription in 63% of opioid-related deaths [34].

### Clinical practice guidelines for the prescription of opioid medication

Clinical practice guidelines (CPGs) have been developed by several countries, including the USA [35] and Canada [2], to support evidence-based prescription of opioid medication for the management of chronic non-cancer pain. A systematic review of opioid prescribing CPGs identified 13 guidelines reporting on recommendations for the prescription of opioids for chronic pain between 2007 and 2013 [36]. While there is between-guideline variability, clinical practice guidelines consistently recommend that HCPs (1) avoid doses ≥ 90–200 mg per day, (2) acquire additional training prior to prescribing methadone, (3) recognize risk of fentanyl patches, (4) titrate cautiously, and (5) reduce doses by at least 25 to 50% when switching opioid [36]. Based on expert consensus rather than rigorous supporting evidence, CPGs also regularly support the use of risk assessment tools, written treatment agreements, and urine drug tests to mitigate risks.

### Clinical inertia in the context of prescribing opioids for chronic pain management

Despite their widespread availability and strong evidence supporting the benefits of their use [37–39], there is a long history of poor uptake of CPGs for chronic disease management by HCPs, with many studies reporting rates of non-adherence at or exceeding 50% [40–42]. HCP non-adherence to CPGs is increasingly referred to as “clinical inertia” [43]. Practically speaking, clinical inertia refers to a HCP’s decision not to initiate, intensify, titrate, or stop treatment despite an indication and recognition of the need to do so. Clinical inertia has most commonly been studied within the context of managing chronic diseases, such as diabetes, cardiovascular disease, hypertension, and dyslipidemia. Within this area, it has been estimated that clinical inertia is responsible for up to 80% of myocardial infarctions and strokes within the context of sub-optimally treated hypertension, diabetes, and dyslipidemia [44].

Our team recently published a review of clinical inertia in the context of chronic disease management where we elucidated the factors associated with this behavior [45]. In brief, clinical inertia is influenced by HCP factors (e.g., knowledge, agreement with guidelines, cognitive biases, motivation), patient factors (e.g., sociodemographics, medical history, lifestyle factors, treatment adherence), and system factors (e.g., time constraints, resources, setting) [45]. System- and patient-level interventions are often mistakenly perceived as being more important or resulting in greater benefit than HCP-level interventions. This protocol will focus on HCP-related factors given that HCP influences (1) account for 50% of variability in clinical inertia [44], (2) are less well understood than system- and patient-factors, (3) often require more sophisticated behavioral corrective interventions, (4) are particularly relevant given the difficult balance between under-prescribing within the context of pain management and over-prescribing within the context of aberrant use, and (5) partially address a priority identified by patients and clinicians who have taken part in a national chronic pain research priority setting process [46].

It is difficult to estimate the impact of clinical inertia on pain, opioid-related morbidity, and mortality because of the relative paucity of available data. Consistent with other chronic diseases, the available evidence suggests that HCP adherence to opioid prescribing guidelines is less than optimal [47]. For example, a survey of more than 200 physicians in Wisconsin reported that only 38% were aware of at least one clinical practice guideline for prescribing opioids in the management of chronic pain [48]. Similarly, an assessment of HCP behavior in a sample of 1300 physicians and residents in Massachusetts reported only partial compliance with national opioid prescribing guidelines (e.g., 43% had a controlled substance agreement, 34% provided > 2 early refills, 63% utilized urine drug tests) [49].

There are many reasons why HCPs deviate from CPGs. One obvious reason is insufficient knowledge. Many HCPs are unaware that evidence of long-term effectiveness for opioids is lacking [4] or that risks include hyperalgesia [50], androgen deficiency [51], and serious fractures from falls [52]. A study of more than 700 family physicians in Canada reported that only 40% of physicians correctly answered two of nine questions pertaining to knowledge of opioid prescribing [53]. Inadequate training and a consequent lack of self-efficacy (i.e., a belief in one’s own ability to personally affect change) are other reasons. More than 70% of 636 family physicians in the province of Quebec surveyed did not feel confident that they could properly prescribe opioids for chronic non-cancer pain, despite 75% of the sample having received continuing education on the topic within the previous year [54]. A similar study observed that 54% of primary care providers surveyed in Massachusetts did not feel sufficiently trained to prescribe opioids [55]. Large volume and inaccessibility of guidelines also contribute to HCP deviation from CPGs. A qualitative study of 12 pain physicians in Ontario identified excessive length and poor formatting as a deterrent to the implementation of the 2010 CPGs for the safe and effective use of opioids for chronic non-cancer pain [56]. An additional reason is fear; nearly 50% of 226 physicians surveyed in Wisconsin reported altering opioid prescribing practices (e.g., limiting refills, lowering dose, reducing prescribed quantity) due to fear of investigation by regulatory agencies [48]. This is also problematic as it may lead to under-treatment of pain or use of an inappropriate opioid tapering schedule contributing to unnecessary suffering, such as aggressive tapering.

In summary, it is safe to assume that adherence to the current guidelines for the prescription of opioid medication for the management of chronic non-cancer pain will make no exception to the general observation that adherence to recommendations detailed in CPG is suboptimal, and would therefore benefit from interventions to improve adherence.

### Objective of the proposed systematic review

To date, there has been no literature synthesis pertaining to factors associated with HCP clinical inertia in the context of prescribing opioid medication for chronic non-cancer pain. A systematic review of this nature is needed for at least four reasons:

1. To elucidate the factors associated with prescribing opioid analgesics in accordance with CPGs.
2. To explore the relative effectiveness of interventions intended to improve the uptake of CPG recommendations for the prescription of opioids to manage chronic non-cancer pain.
3. To guide the development of novel interventions to optimizing the prescription of opioid analgesics.
4. To identify gaps in knowledge pertaining to clinical inertia for the prescription of opioid analgesics.

## Methods

### Protocol reporting and registration

This review protocol was prepared in accordance with the Preferred Reporting Items for Systematic Review and Meta-Analyses Protocol (PRISMA-P) guidelines [57] (refer to Additional file 1). The protocol is registered with Prospective Register of Systematic Reviews (PROSPERO; registration#CRD42018091640).

### Structured clinical question(s)

1. What are the knowledge, attitudes, beliefs, and practices that HCPs hold regarding the prescription of opioids for chronic non-cancer pain?
2. Do knowledge, attitudes, beliefs, and practices pertaining to prescribing opioids for the management of chronic non-cancer pain differ by HCP characteristics (e.g., sex, discipline, duration of practice) or patient factors (e.g., sex, history of addiction)?
3. What is the prevalence of clinical inertia for prescribing opioids for the management of chronic non-cancer pain?
4. Have interventions been developed that have an impact on opioid prescribing behavior for the management of chronic non-cancer pain?

### Data sources and search strategy

A preliminary search strategy will be created under the guidance of an experienced information specialist (BS; refer to Additional file 2). A second information specialist will peer-review the strategy prior to execution using the Peer Review for Electronic Search Strategies (PRESS) checklist [58]. Using the OVID platform, we will search Ovid MEDLINE® (1946 to April 23, 2018), including Epub Ahead of Print and In-Process & Other Non-Indexed Citations, Embase Classic + Embase (1947 to April 23, 2018), and PsycINFO (1806 to April 23, 2018). We will also search the Cochrane Library on Wiley and CINAHL (1981 to present) on Ebsco. The Joanna Briggs Institute EBP Database and PROSPERO (2011 to present) will be searched for completed systematic reviews.

Search strategies will incorporate controlled vocabulary for relevant themes (e.g., “Analgesics,” “Opioid,” “Health Personnel,” “Guideline Adherence”) and keywords (e.g., “opioid,” “physician,” “clinical inertia”). Vocabulary and syntax will be adjusted across databases. No language restriction will be imposed, but where possible, animal-only studies will be removed from the results. No study-specific filters will be applied given the wide range of study designs of interest.

### Study eligibility criteria

#### Population

Our population of interest is HCPs who prescribe opioid medication (e.g., physician, dentist, nurse practitioner). Studies that include medical residents will also be eligible for inclusion.

#### Interest

Clinical inertia (i.e., barriers and facilitators of adherence to long-term opioid prescribing guidelines) for the management of chronic non-cancer pain. We also aim to capture interventions that have been conducted to improve adherence to opioid prescribing guidelines within the context of chronic pain.

#### Context

Primary and specialist care of patients with chronic non-cancer pain in North America.

#### Outcomes

HCP knowledge, attitudes, beliefs, and practices pertaining to opioid prescribing guidelines for the management of chronic non-cancer pain. HCP behavior will be coded for clinical inertia.

#### Designs

A range of study designs will be eligible. Survey and epidemiological, qualitative, and uncontrolled studies reporting on facilitators and barriers towards implementing long-term opioid prescribing guidelines will be eligible given that such research can maximize the value of a systematic review to inform clinical practice, policy, and decision-making [59, 60]. Controlled studies, experimental designs, non-randomized controlled studies, and retrospective and prospective cohort studies that include a control group, including before-after studies, will be eligible for inclusion in order to evaluate the efficacy/effectiveness of interventions designed to improve adherence to long-term opioid prescribing guidelines.

The following inclusion criteria will be applied:

- Study involves the prescription of opioid medication for adults (≥ 18 years of age). We chose to focus on adults given that comparable guidelines have not been published for children and youth [61]. As such, practice and clinical inertia would likely be quite different for children/youth compared to adults.
- The study concerned at least one of the following: knowledge, attitudes, beliefs, or practices pertaining to long-term opioid prescribing for the management of chronic non-cancer pain.
- The study reported data from HCPs who prescribes opioid medication (e.g., physician, specialist (MD), dentist, pharmacist, nurse practitioner). Studies that include medical residents are also considered eligible for inclusion.
- The study reports on original data. Multiple reports of single studies will be handled avoiding duplication of source data.
- The study was conducted in North America. We chose this distinction because opioid-related prescribing practices, non-medical use, and harms are greater in North America than anywhere else in the world [62]. Differences in the organization of health systems, prescription practices, dispensing and medical cultures, and patient expectations have been proposed as factors that contribute to the differences observed [62].

The following exclusion criteria will be applied:

- The primary focus of the study pertains to non-medical opioid use (i.e., opioid abuse and dependence).
- The primary focus of the study pertains to the prescription of medication to prevent or manage non-medical opioid use (e.g., naloxone, suboxone, methadone). Studies focusing on the prescription of these medications will be eligible if they are prescribed specifically for pain management.
- Conference abstracts.

### Screening and data extraction

Two trained graduate-level research assistants will independently screen titles and abstracts of all identified search results for potential inclusion. The reviewers will select all potentially relevant citations reporting on HCP knowledge, attitudes, beliefs, or practices regarding prescribing opioid medication for chronic non-cancer pain. In doubt, references will be included at the title and abstract level. Full-text publications of all potentially relevant articles, selected by either reviewer, will be retrieved and examined for eligibility. The reference management software Rayyan [63] will be used to remove duplicates and sort inclusions and exclusions. Agreement between reviewers will be quantified using the Kappa statistic [64] before proceeding to solve disagreements. Disagreements on inclusion and exclusion of articles between reviewers will be resolved by consensus or arbitration by a third reviewer (JAR) if necessary. The study selection process will be documented using a PRISMA flow diagram [65].

In duplicate, the same two reviewers will independently extract information from all potentially eligible studies using a pre-designed data extraction Excel spreadsheet. Interpreters will be enlisted to screen and translate non-English language studies as required. Two levels of extraction will occur. Limited extraction will be implemented for all articles that were identified as potentially eligible after the initial screening of titles and abstracts for inclusion and exclusion criteria and will include the following:

1. Journal article information (i.e., first author, journal, publication year).
2. Basic screening of inclusion and exclusion criteria as outlined in the “Study eligibility criteria” section.

The reviewers will then categorize the articles as “in” or “out” and list the reason for exclusion. Excluded articles will be listed in the PRISMA diagram, grouped by reasons for exclusion.

Following the brief extraction, full-data extraction of included articles will be completed independently by each reviewer. The following information will be extracted for each article:

1. Information on methodology (i.e., study design, length of follow-up, country, incentives offered)
2. Participant information (i.e., total sample size, recruitment method, defined sub-groups, provider discipline)
3. Details on the measures used (i.e., instruments used, intervention characteristics, method of delivery)
4. Outcomes: Similar to other systematic reviews of physician beliefs [66], knowledge, attitudes, beliefs, and practice will be extracted verbatim and coded in accordance with the 14 domains defined by the Theoretical Domains Framework [67, 68]. Inter-rater reliability of these categories will be assessed using the Kappa statistic [64].
5. Results of the study (e.g., response rate, missing data, handling of missing data, sub-groupings, proportion of HCPs endorsing beliefs along with range and confidence intervals).
6. Risk of bias. Risk of bias will be collected using validated tools, refer to the “Risk of bias and quality assessment” section.

Once completed, the full extractions will be compared across raters to ensure accuracy. Discrepancies will be resolved by an independent arbiter (JAR).

### Risk of bias and quality assessment

The methodological quality and risk of bias of included studies will be assessed independently and in duplicate by the same two trained research assistants. Risk of bias in randomized trials will be assessed using a modified version [69] of the Cochrane risk of bias tool [70]. The domains assessed include random sequence generation, allocation concealment, blinding of participants and personnel, attrition, reporting bias, and other sources of bias. Each domain will be assigned a judgment of “high risk of bias” or “low risk of bias” [70]. Corresponding authors of studies assessed will be contacted for clarification when insufficient evidence is reported to assess risk of bias. No study-level summary judgment of risk of bias will be performed given that such summative judgments do not correspond with treatment outcomes [71].

Methodological quality of cross-sectional studies will be assessed using the “instrument for risk of bias in cross-sectional studies of attitudes and practices” available from Evidence Partners and contributed by the Clinical Advances Through Research and Information Translation (CLARITY) Group at McMaster University [72]. Five domains are assessed for cross-sectional survey studies, including representativeness of sample to the population of interest, adequacy of response rate, missing data, clinical sensibility, and reliability and validity of the instrument used. Each domain is assigned a judgment of “definite risk of bias,” “probable risk of bias,” “probable risk of low bias,” or “definite risk of low bias.” A table will be constructed that depicts risk of bias.

Methodological quality of cohort studies will be assessed using Joanna Briggs Institute checklist for cohort studies [73]. Eleven domains are assessed, including similarity of groups recruited, timing of exposure, validity and reliability of exposure, identification of confounding factors, mitigation of confounds, validity and reliability of outcome measured, adequacy of follow-up, completeness of follow-up, appropriateness of statistical analyses. Each domain is assigned a rating of “yes,” “no,” “unclear,” or “not applicable.” A table will be constructed that depicts risk of bias.

Confidence in information obtained from qualitative studies will be assessed in accordance with recommendations made by the Cochrane Qualitative and Implementation Methods Group [74] using a multi-dimensional concept of quality that includes (1) clarity of aims and research question, (2) congruence between questions and methods, (3) rigor of sampling and data collection, and (4) overall conceptual depth and breadth are reflected in study design, process, and results. As recommended [74], information will be collected by having two independent raters appraise study quality using the 10-item Critical Appraisal Skills Programme (CASP; [75]) quality assessment tool for qualitative studies. Each item will be rated as “yes,” “no,” or “unclear.” Quality assessment will be depicted using a table.

### Approach to evidence synthesis

Included studies will be categorized according to study design. Frequencies and percentages will be reported for categorical variables. Means and standard deviations (SDs) or median and interquartile range will be reported for continuous data as appropriate. The extracted proportions of studies that include the same category of knowledge, beliefs, attitudes, and practices will be pooled. A Q-test adapted for proportions will be used to test for heterogeneity of proportions [64]. As recommended [64], random effects models will be performed to account for the imperfect measurement of knowledge, beliefs, attitudes, and practices. The proportions from each study will be weighted by the size of the respective sample. If a single study uses several items to measure the same underlying construct (e.g., “prescribing guidelines are developed using populations that are not representative of the chronic pain presentations that I see in my practice”), then the most commonly used item will be utilized. Results will be presented as pooled proportions, 95% confidence intervals, range, and pooled frequencies for each category of knowledge, attitudes, beliefs, and practices. If sufficient data is obtained, results will be broken down by HCP characteristics (e.g., sex, discipline) to perform sub-group analysis. Sensitivity analyses will be performed on studies that are deemed “low risk of bias.”

If studies are identified that evaluate the effect of an intervention to change HCP adherence to recommendations in CPGs for the prescription of opioid medication, relevant statistics (e.g., F-values, means and SDs) will be used to calculate standardized mean differences using formulae described previously [76]. A meta-analysis will be performed if ≥ 3 studies are identified that evaluate a theoretically and methodologically similar intervention [77]. Analytic computations will be performed using Comprehensive Meta-Analysis software (CMA; [78]). Evidence for publication bias will be assessed through visual inspection of a funnel plot, and fail-safe Ns will be calculated using Orwin’s formula [79] with the recommended criterion of effect size of 0.20.

### Assessing confidence in evidence

As per recommendations [74], the Grades of Recommendation, Assessment, Development, and Evaluation-Confidence in the Evidence from Qualitative Reviews (GRADE-CERQual) approach [80] will be used to assess confidence in synthesized qualitative results. The CERQual approach includes four components: (1) methodological limitations of individual studies, (2) relevance of the review question of individual studies, (3) coherence of review results, and (4) adequacy of data supporting a review result. Confidence in quantitative evidence will be evaluated using the GRADE approach [81].

## Discussion

This will be the first knowledge synthesis to elucidate the factors associated with clinical inertia with respect to HCP guideline adherence for the safe prescription of opioid medication for the management of chronic non-cancer pain. The knowledge generated by this synthesis is vital for better understanding the concerns that HCPs have about prescribing opioid medications. Such knowledge will be used to elucidate theory-driven and evidence-based behavior change principles that specifically target the various facets of clinical inertia identified. Identified behavior change principles can be integrated into existing interventions that have proven effective (e.g., education) or used to develop novel interventions (e.g., education delivered using motivational communication—a broad set of evidence-based, patient-centered techniques designed to promote motivation for behavior change [82]). The ultimate goal of this work is to optimize opioid prescribing practices in order to prevent opioid-related morbidity and mortality without restricting a HCP’s ability to select the most appropriate treatment for an individual patient.

## Additional files

Additional file 1: Preferred Reporting Items for Systematic Review and Meta-Analyses Protocol (PRISMA-P) checklist. (DOCX 32 kb)

Additional file 2: Sample search strategy. (DOCX 15 kb)

## Abbreviations

CASP: Critical Appraisal Skills Programme
CIHR: Canadian Institutes of Health Research
CPG: Clinical practice guideline
HCP: Healthcare provider
PRESS: Peer Review of Electronic Search Strategies
PRISMA-P: Preferred Reporting Items for Systematic Review and Meta-Analyses Protocol
PROSPERO: Prospective Register of Systematic Reviews

## References

[1] Lee M, Silverman SM, Hansen H, Patel VB, Manchikanti L (2011). A comprehensive review of opioid-induced hyperalgesia. Pain Physician.

[2] Busse JW, Craigie S, Juurlink DN, Buckley DN, Wang L, Couban RJ, Agoritsas T, Akl EA, Carrasco-Labra A, Cooper L (2017). Guideline for opioid therapy and chronic noncancer pain. CMAJ.

[3] Chou R, Turner JA, Devine EB, Hansen RN, Sullivan SD, Blazina I, Dana T, Bougatsos C, Deyo RA (2015). The effectiveness and risks of long-term opioid therapy for chronic pain: a systematic review for a National Institutes of Health Pathways to Prevention Workshop. Ann Intern Med.

[4] Kolodny A, Courtwright DT, Hwang CS, Kreiner P, Eadie JL, Clark TW, Alexander GC (2015). The prescription opioid and heroin crisis: a public health approach to an epidemic of addiction. Annu Rev Public Health.

[5] Merskey H (1979). Pain terms: a list with definitions and notes on usage. Recommended by the International Association for the Study of Pain. Pain.

[6] Institute of Medicine (IoM) (2011). Relieving pain in America: a blueprint for transforming prevention, care, education, and research.

[7] Breivik H, Collett B, Ventafridda V, Cohen R, Gallacher D (2006). Survey of chronic pain in Europe: prevalence, impact on daily life, and treatment. Eur J Pain.

[8] Fayaz A, Croft P, Langford RM, Donaldson LJ, Jones GT (2016). Prevalence of chronic pain in the UK: a systematic review and meta-analysis of population studies. BMJ Open.

[9] Hauser W, Wolfe F, Henningsen P, Schmutzer G, Brahler E, Hinz A (2014). Untying chronic pain: prevalence and societal burden of chronic pain stages in the general population - a cross-sectional survey. BMC Public Health.

[10] Nahin RL (2015). Estimates of pain prevalence and severity in adults: United States, 2012. J Pain.

[11] Schopflocher D, Taenzer P, Jovey R (2011). The prevalence of chronic pain in Canada. Pain research and management.

[12] Tsang A, Von Korff M, Lee S, Alonso J, Karam E, Angermeyer MC, Borges GL, Bromet EJ, Demytteneare K, de Girolamo G (2008). Common chronic pain conditions in developed and developing countries: gender and age differences and comorbidity with depression-anxiety disorders. J Pain.

[13] McBeth J, Jones K (2007). Epidemiology of chronic musculoskeletal pain. Best Pract Res Clin Rheumatol.

[14] Hadjistavropoulos T, Marchildon GP, Fine PG, Herr K, Palley HA, Kaasalainen S, Beland F (2009). Transforming long-term care pain management in North America: the policy-clinical interface. Pain Med.

[15] Gourlay DL, Heit HA, Almahrezi A (2005). Universal precautions in pain medicine: a rational approach to the treatment of chronic pain. Pain Med.

[16] Portenoy RK, Foley KM (1986). Chronic use of opioid analgesics in non-malignant pain: report of 38 cases. Pain.

[17] Porter J, Jick H (1980). Addiction rare in patients treated with narcotics. N Engl J Med.

[18] Leung PTM, Macdonald EM, Stanbrook MB, Dhalla IA, Juurlink DN (2017). A 1980 letter on the risk of opioid addiction. N Engl J Med.

[19] Cheatle MD (2015). Prescription opioid misuse, abuse, morbidity, and mortality: balancing effective pain management and safety. Pain Med.

[20] Dart RC, Severtson SG, Bucher-Bartelson B (2015). Trends in opioid analgesic abuse and mortality in the United States. N Engl J Med.

[21] Canadian Institute for Health Informatics (CIHI) (2017). Pan-Canadian trends in the prescription of opioids, 2012 to 2016.

[22] Jones C (2013). Trends in the distribution of selected opioids by state, US, 1999–2011. National Meeting, safe state Alliance.

[23] Center for Disease Control & Prevention (CDC) (2011). Vital signs: overdoses of prescription opioid pain relievers---United States, 1999--2008. MMWR Morbidity and mortality weekly report.

[24] Drug abuse warning network. National estimates of drug-related emergency department visits. 2011. Rockville (MD): Substance Abuse and Mental Health Services Administration; 2013. Report no.: DAWN Series D-39, HHS Publication No. (SMA) 13-4760.

[25] Drug Abuse Warning Network (2011). National estimates of drug-related emergency department visits. 2011. Substance abuse and mental health service administration.

[26] Chen LH, Hedegaard H, Warner M (2014). Drug-poisoning deaths involving opioid analgesics: United States, 1999-2011**.** NCHS data brief.

[27] Rudd RA, Seth P, David F, Scholl L (2016). Increases in drug and opioid-involved overdose deaths - United States, 2010-2015. MMWR Morb Mortal Wkly Rep.

[28] Apparent opioid-related deaths [https://www.canada.ca/en/health-canada/services/substance-abuse/prescription-drug-abuse/opioids/apparent-opioid-related-deaths.html]. Accessed 18 Oct 2017.

[29] European Monitoring Center for Drugs and Addictions (2016). European drug report 2016.

[30] Casati A, Sedefov R, Pfeiffer-Gerschel T (2012). Misuse of medicines in the European Union: a systematic review of the literature. Eur Addict Res.

[31] Shei A, Hirst M, Kirson NY, Enloe CJ, Birnbaum HG, Dunlop WC (2015). Estimating the health care burden of prescription opioid abuse in five European countries. Clinicoecon Outcomes Res.

[32] Compton WM, Jones CM, Baldwin GT (2016). Relationship between nonmedical prescription-opioid use and heroin use. N Engl J Med.

[33] King NB, Fraser V, Boikos C, Richardson R, Harper S (2014). Determinants of increased opioid-related mortality in the United States and Canada, 1990-2013: a systematic review. Am J Public Health.

[34] Dhalla IA, Mamdani MM, Gomes T, Juurlink DN (2011). Clustering of opioid prescribing and opioid-related mortality among family physicians in Ontario. Can Fam Physician.

[35] Dowell D, Haegerich TM, Chou R (2016). CDC guideline for prescribing opioids for chronic pain—United States, 2016. Jama.

[36] Nuckols TK, Anderson L, Popescu I, Diamant AL, Doyle B, Di Capua P, Chou R (2014). Opioid prescribing: a systematic review and critical appraisal of guidelines for chronic pain. Ann Intern Med.

[37] Carlhed R, Bojestig M, Wallentin L, Lindstrom G, Peterson A, Aberg C, Lindahl B (2006). Improved adherence to Swedish national guidelines for acute myocardial infarction: the Quality Improvement in Coronary Care (QUICC) study. Am Heart J.

[38] Grimshaw JM, Russell IT (1993). Effect of clinical guidelines on medical practice: a systematic review of rigorous evaluations. Lancet.

[39] Lugtenberg M, Burgers JS, Westert GP (2009). Effects of evidence-based clinical practice guidelines on quality of care: a systematic review. Qual Saf Health Care.

[40] Balder JW, Scholtens S, de Vries JK, van Schie LM, Boekholdt SM, Hovingh GK, Kamphuisen PW, Kuivenhoven JA (2015). Adherence to guidelines to prevent cardiovascular diseases: the LifeLines cohort study. Neth J Med.

[41] McGlynn EA, Asch SM, Adams J, Keesey J, Hicks J, DeCristofaro A, Kerr EA (2003). The quality of health care delivered to adults in the United States. N Engl J Med.

[42] Sager HB, Linsel-Nitschke P, Mayer B, Lieb W, Franzel B, Elsasser U, Schunkert H (2010). Physicians’ perception of guideline-recommended low-density lipoprotein target values: characteristics of misclassified patients. Eur Heart J.

[43] Phillips LS, Branch WT, Cook CB, Doyle JP, El-Kebbi IM, Gallina DL, Miller CD, Ziemer DC, Barnes CS (2001). Clinical inertia. Ann Intern Med.

[44] O'Connor PJ, Sperl-Hillen JM, Johnson PE, Rush WA, Biltz G (2005). Clinical inertia and outpatient medical errors.

[45] Lavoie KL, Rash JA, Campbell TS (2017). Changing provider behavior in the context of chronic disease management: focus on clinical inertia. Annu Rev Pharmacol Toxicol.

[46] Poulin Patricia, Shergill Yaadwinder, Romanow Heather, Busse Jason W., Chambers Christine T., Cooper Lynn, Forgeron Paula A., Olsen Harper Anita, Hudspith Maria, Iorio Alfonso, Lalloo Chitra, Ouellette Carley, Robertson Rosalind, Smeenk Sandy, Stevens Bonnie, Stinson Jennifer (2018). Researching what matters to improve chronic pain care in Canada: A priority-setting partnership process to support patient-oriented research. Canadian Journal of Pain.

[47] Victor TW, Alvarez NA, Gould E (2009). Opioid prescribing practices in chronic pain management: guidelines do not sufficiently influence clinical practice. J Pain.

[48] Wolfert MZ, Gilson AM, Dahl JL, Cleary JF (2010). Opioid analgesics for pain control: Wisconsin physicians’ knowledge, beliefs, attitudes, and prescribing practices. Pain Med.

[49] Khalid L, Liebschutz JM, Xuan Z, Dossabhoy S, Kim Y, Crooks D, Shanahan C, Lange A, Heymann O, Lasser KE (2015). Adherence to prescription opioid monitoring guidelines among residents and attending physicians in the primary care setting. Pain Med.

[50] Angst MS, Clark JD (2006). Opioid-induced hyperalgesia: a qualitative systematic review. Anesthesiology.

[51] Smith HS, Elliott JA (2012). Opioid-induced androgen deficiency (OPIAD). Pain Physician.

[52] Saunders KW, Dunn KM, Merrill JO, Sullivan M, Weisner C, Braden JB, Psaty BM, Von Korff M (2010). Relationship of opioid use and dosage levels to fractures in older chronic pain patients. J Gen Intern Med.

[53] Allen MJ, Asbridge MM, Macdougall PC, Furlan AD, Tugalev O (2013). Self-reported practices in opioid management of chronic noncancer pain: a survey of Canadian family physicians. Pain Res Manag.

[54] Roy E, Cote RJ, Hamel D, Dube PA, Langlois E, Labesse ME, Thibault C, Boulanger A (2017). Opioid prescribing practices and training needs of Quebec family physicians for chronic noncancer pain. Pain Res Manag.

[55] Jamison R, KA S, E S, Ross E (2014). Beliefs and attitudes about opioid prescribing and chronic pain management: survey of primary care providers. Journal of opioid management.

[56] Chang Y, Zhu KL, Florez ID, Cho SM, Zamir N, Toma A, Mirza RD, Guyatt GH, Buckley N, Busse JW (2016). Attitudes toward the Canadian guideline for safe and effective use of opioids for chronic non-cancer pain: a qualitative study. J Opioid Manag.

[57] Moher D, Shamseer L, Clarke M, Ghersi D, Liberati A, Petticrew M, Shekelle P, Stewart LA (2015). Preferred reporting items for systematic review and meta-analysis protocols (PRISMA-P) 2015 statement. Syst Rev.

[58] McGowan J, Sampson M, Salzwedel DM, Cogo E, Foerster V, Lefebvre C (2016). PRESS peer review of electronic search strategies: 2015 guideline statement. J Clin Epidemiol.

[59] Mays N, Pope C, Popay J (2005). Systematically reviewing qualitative and quantitative evidence to inform management and policy-making in the health field. J Health Serv Res Policy.

[60] Popay J (2005). Moving beyond floccinaucinihilipilification: enhancing the utility of systematic reviews. J Clin Epidemiol.

[61] Schechter NL, Walco GA (2016). The potential impact on children of the CDC guideline for prescribing opioids for chronic pain: above all, do no harm. JAMA Pediatr.

[62] Fischer B, Keates A, Buhringer G, Reimer J, Rehm J (2014). Non-medical use of prescription opioids and prescription opioid-related harms: why so markedly higher in North America compared to the rest of the world?. Addiction.

[63] Ouzzani M, Hammady H, Fedorowicz Z, Elmagarmid A (2016). Rayyan-a web and mobile app for systematic reviews. Syst Rev.

[64] Egger M, Smith GD, Altman D. Systematic reviews in health care: meta-analysis in context (2nd Ed.). London: BMJ Publishing Group; 2001.

[65] Moher D, Liberati A, Tetzlaff J, Altman DG (2009). Preferred reporting items for systematic reviews and meta-analyses: the PRISMA statement. Ann Intern Med.

[66] Vogt F, Hall S, Marteau TM (2005). General practitioners’ and family physicians’ negative beliefs and attitudes towards discussing smoking cessation with patients: a systematic review. Addiction.

[67] Cane J, O'Connor D, Michie S (2012). Validation of the theoretical domains framework for use in behaviour change and implementation research. Implement Sci.

[68] Atkins L, Francis J, Islam R, O'Connor D, Patey A, Ivers N, Foy R, Duncan EM, Colquhoun H, Grimshaw JM (2017). A guide to using the Theoretical Domains Framework of behaviour change to investigate implementation problems. Implement Sci.

[69] Akl EA, Sun X, Busse JW, Johnston BC, Briel M, Mulla S, You JJ, Bassler D, Lamontagne F, Vera C (2012). Specific instructions for estimating unclearly reported blinding status in randomized trials were reliable and valid. J Clin Epidemiol.

[70] Higgins JP, Altman DG, Gotzsche PC, Juni P, Moher D, Oxman AD, Savovic J, Schulz KF, Weeks L, Sterne JA (2011). The Cochrane Collaboration’s tool for assessing risk of bias in randomised trials. BMJ.

[71] Dechartres A, Altman DG, Trinquart L, Boutron I, Ravaud P (2014). Association between analytic strategy and estimates of treatment outcomes in meta-analyses. JAMA.

[72] Risk of bias intrument for cross-sectional surveys of attitudes and practices [https://www.evidencepartners.com/wp-content/uploads/2017/09/Risk-of-Bias-Instrument-for-Cross-Sectional-Surveys-of-Attitudes-and-Practices.pdf].

[73] Moola S, Munn Z, Tufanaru C, Aromataris E, Sears K, Sfetcu R, Currie M, Qureshi R, Mattis P, Lisy K, Aromataris EM, Adelaide Z (2017). Chapter 7: systematic reviews of etiology and risk. Joanna Briggs Institute reviewer’s manual.

[74] Noyes J, Booth A, Flemming K, Garside R, Harden A, Lewin S, Pantoja T, Hannes K, Cargo M, Thomas J: Cochrane Qualitative and Implementation Methods Group guidance paper 3: methods for assessing methodological limitations, data extraction and synthesis, and confidence in synthesized qualitative findings**.** *J Clin Epidemiol* 2018**:**10.1016/j.jclinepi.2017.1006.1020. 10.1016/j.jclinepi.2017.06.020 29247700

[75] Making sense of evidence: 10 questions to help you make sense of qualitative research [http://media.wix.com/ugd/dded87_29c5b002d99342f788c6ac670e49f274.pdf]. Accessed 13 Mar 2018.

[76] Lipsey MW, Wilson DB. Practical meta-analysis. Thousand Oaks: Sage Publications, Inc; 2001.

[77] Valentine JC, Pigott TD, Rothstein HR (2010). How many studies do you need? A primer on statistical power for meta-analysis. J Educ Behav Stat.

[78] Borenstein M, Hedges LV, Higgins JP, Rothstein HR (2015). Biostat: comprehensive meta-analysis (version 3)[software].

[79] Orwin RG (1983). A fail-safe N for effect size in meta-analysis. J Educ Stat.

[80] Thomas J, Harden A, Oakley A, Oliver S, Sutcliffe K, Rees R, Brunton G, Kavanagh J (2004). Integrating qualitative research with trials in systematic reviews. BMJ.

[81] Guyatt GH, Oxman AD, Vist GE, Kunz R, Falck-Ytter Y, Alonso-Coello P, Schunemann HJ (2008). GRADE: an emerging consensus on rating quality of evidence and strength of recommendations. BMJ.

[82] Rouleau CR, Lavoie KL, Bacon SL, Vallis M, Corace K, Campbell TS (2015). Training healthcare providers in motivational communication for promoting physical activity and exercise in cardiometabolic health settings: do we know what we are doing?. Current Cardiovascular Risk Reports.

