# Supplementary material for: Healthcare provider knowledge, attitudes, beliefs, and practices surrounding the prescription of opioids for chronic non-cancer pain in North America: protocol for a mixed-method systematic review
Source: Syst Rev. 2018 Nov 13;7:189. doi: 10.1186/s13643-018-0858-7 (PMC6234680; doi:10.1186/s13643-018-0858-7)
Supplement: Supplementary file 2 — Sample search strategy. (DOCX 15 kb) [file 13643_2018_858_MOESM2_ESM.docx]

Opioids – OCKS

Draft Strategy

2018 Apr 2

MEDLINE

Database: Ovid MEDLINE(R) Epub Ahead of Print, In-Process & Other Non-Indexed Citations, Ovid MEDLINE(R) Daily and Ovid MEDLINE(R) <1946 to Present>

Search Strategy:

--------------------------------------------------------------------------------

1 Analgesics, Opioid/ (36270)

2 (opioid? or opiate?).tw,kf. (91483)

3 exp Buprenorphine/ (4535)

4 (buprenorphine or anorfin$2 or belbuca$2 or buprenex$2 or buprex$2 or buprine$2 or butrans$2 or finibron$2 or lepetan$2 or nih 8805 or nih8805 or norphin$2 or pentorel$2 or prefin$2 or probuphine$2 or rx 6029 m or rx 6029m or rx6029m or subutex$2 or temgesic$2 or transtec$2 or um 952 or um952).tw,kf. (5788)

5 (buprenorphine-naloxone or naloxone-buprenorphine or suboxone$2 or zubsolv$2).tw,kf. (599)

6 exp Fentanyl/ (14712)

7 (fentanyl or alfentanil$2 or alfenta$2 or alfentanyl$2 or beta hydroxymefentanyl or brifentanil$2 or carfentanil$2 or duragesic$2 or fanaxal$2 or fentanest$2 or fentora$2 or hypnorm$2 or limifen$2 or lofentanil$2 or mefentanyl$2 or mirfentanil$2 or ocfentanil$2 or phentanyl$2 or R-39209 or R-4263 or rapifen$2 or remifentanil$2 or sublimaze$2 or sufenta$2 or sufentanil$2 or sulfentanyl$2 or trefentanil$2).tw,kf. (23937)

8 Hydrocodone/ (530)

9 (hydrocodone or bekadid$2 or codinovo$2 or dico$2 or dicodid$2 or dihydrocodeinone$2 or hycodan$2 or hycon$2 or hydrocodeinonebitartrate$2 or hydrocodon$2 or hydrocon$2 or hydrocodonum$2 or robidone$2).tw,kf. (5604)

10 Hydromorphone/ (1162)

11 (hydromorphon* or biomorphyl$2 or cofalaudid$2 or dihydromorphinon* or dihydromorphon* or diladid$2 or dilaudid$2 or dimorphon* or dolonovag$2 or exalgo$2 or hydal$2 or hydromorph contin$2 or hydromorphinone$2 or hydrostat$2 or hymorphan$2 or jurnista$2 or laudaconum$2 or novolaudon$2 or opidol$2 or paliadon retardkaps$2 or palladon$2 or rexaphon$2 or semcox$2 or sophidone$2).tw,kf. (11435)

12 exp Methadone/ (11567)

13 (methadone or adanon$2 or adanon hydrochloride$2 or algidon$2 or algolysin$2 or algoxale$2 or althose$2 or althose hydrochloride$2 or amidon$2 or amidona$2 or amidone$2 or amidosan$2 or an 148 or an148 or anadon$2 or biodone$2 or butalgin$2 or deamin$2 or depridol$2 or diaminon$2 or dianone$2 or dolafin$2 or dolamid$2 or dolesone$2 or dolmed$2 or dolophine$2 or dolophine hydrochloride$2 or dorex$2 or dorexol$2 or eptadone$2 or fenadon$2 or gobbidona$2 or heptadon$2 or heptanon$2 or hoe 10820 or hoe10820 or ketalgin$2 or l polamidon$2 or mecodin$2 or mepecton$2 or mephenon$2 or metadol$2 or metadon$2 or metasedin$2 or methaddict$2 or methadose$2 or methaforte mix$2 or miadone$2 or moheptan$2 or pallidone$2 or phenadon$2 or physepton$2 or physeptone$2 or polamidon$2 or polamivet$2 or polamivit$2 or sinalgin$2 or symoron$2 or westadone$2).tw,kf. (14391)

14 Morphine/ (36636)

15 (morphine or anpec$2 or duramorph$2 or epimorph$2 or miro$2 or morfin$2 or morfine$2 or morphin$2 or morphinium$2 or morphium$2 or MS contin or morphia$2 or opso$2 or oramorph$2 or SDZ 202-250 or SDZ202-250 or skenan$2 or transmorphine$2 or trans-morphine$2).tw,kf. (49458)

16 Oxycodone/ (1883)

17 (oxycodone or bionine$2 or bionone$2 or bolodorm$2 or broncodal$2 or bucodal$2 or cafacodal$2 or cardanon$2 or codenon$2 or codix 5 or "col 003" or col003 or DETERx$2 or dihydrohydroxycodeinone or dihydrohydroxydodeinone or dihydrone$2 or dinarkon$2 or endone$2 or eubine$2 or eucodal$2 or eucodale$2 or eucodalum$2 or eudin$2 or eukdin$2 or eukodal$2 or eumorphal$2 or eurodamine$2 or eutagen$2 or hydrocodal$2 or hydroxycodeinoma$2 or ludonal$2 or m-oxy or medicodal$2 or narcobasina$2 or narcobasine$2 or narcosin$2 or nargenol$2 or narodal$2 or nsc 19043 or nucodan$2 or opton$2 or ossicodone$2 or oxanest$2 or oxaydo$2 or oxecta$2 or oxicone$2 or oxicontin$2 or oxiconum$2 or oxikon$2 or oxy ir or oxycod$2 or oxycodeinon$2 or oxycodeinonhydrochloride or oxycodone hydrochloride or oxycodonhydrochlorid or oxycodyl$2 or oxycone$2 or oxycontin$2 or oxydose$2 or oxyfast$2 or oxygesic$2 or oxyir$2 or oxykon$2 or oxynorm$2 or pancodine$2 or pavinal$2 or percolone$2 or pronarcin$2 or remoxy$2 or roxicodone$2 or roxycodone$2 or sinthiodal$2 or stupenal$2 or supeudol$2 or tebodal$2 or tekodin$2 or thecodin$2 or theocodin$2 or xtampa$2 or xtampza$2).tw,kf. (2929)

18 or/1-17 [OPIOIDS] (178782)

19 Health Personnel/ (32807)

20 ((health or healthcare or health care) adj (personnel or practitioner? or provider?)).tw,kf. (34101)

21 exp Physicians/ (119185)

22 (doctor? or family clinician? or family practitioner? or general practitioner? or GP or GPs or medical practitioner? or medical specialist? or physician?).tw,kf. (514095)

23 exp Dentists/ (17730)

24 (dentist? or endodontist? or exodontist? or maxillofacial surgeon? or maxillo-facial surgeon? or oral surgeon? or periodontist? or prosthodontist?).tw,kf. (36580)

25 exp Nurses/ (81963)

26 (nurse or nurses).tw,kf. (235872)

27 Pharmacists/ (14086)

28 pharmacist?.tw,kf. (26845)

29 prescriber?.tw,kf. (5542)

30 Primary Health Care/ (66990)

31 (primary adj2 (care or healthcare or health care)).tw,kf. (121890)

32 or/19-31 [HEALTHCARE PROVIDERS/PRIMARY HEALTH CARE] (986606)

33 18 and 32 [OPIOIDS - HEALTHCARE PROVIDERS/PRIMARY HEALTH CARE] (8918)

34 Health Personnel/px [Psychology] (5509)

35 exp Physicians/px [Psychology] (19113)

36 exp Dentists/px [Psychology] (2037)

37 exp Nurses/px [Psychology] (18334)

38 Pharmacists/px [Psychology] (1158)

39 Practice Guidelines as Topic/ (102977)

40 Guideline Adherence/ (27851)

41 ((CPG or CPGs or guideline? or clinical pathway? or policy or policies or practice parameter? or protocol? or recommendation? or standard?) adj2 (adopt* or adher* or effect* or follow* or comply* or complied or complies or complian* or incorporat* or uptake)).tw,kf. (53275)

42 (institution* adj2 (adher* or follow* or comply* or complied or complies or complian* or uptake)).tw,kf. (2037)

43 (provider? adj2 (adher* or follow* or comply* or complied or complies or complian* or uptake)).tw,kf. (1113)

44 "Health Knowledge, Attitudes, Practice"/ (94534)

45 Attitudes/ (43928)

46 exp Attitude of Health Personnel/ (143384)

47 Attitude to Death/ (15049)

48 Attitude to Health/ (79711)

49 ((doctor? or family clinician? or family practitioner? or general practitioner? or GP or GPs or medical practitioner? or medical specialist? or physician? or dentist? or endodontist? or exodontist? or maxillofacial surgeon? or maxillo-facial surgeon? or oral surgeon? or periodontist? or prosthodontist? or nurse or nurses or pharmacist? or prescriber? or provider?) adj3 attitud*).tw,kf. (10381)

50 ((doctor? or family clinician? or family practitioner? or general practitioner? or GP or GPs or medical practitioner? or medical specialist? or physician? or dentist? or endodontist? or exodontist? or maxillofacial surgeon? or maxillo-facial surgeon? or oral surgeon? or periodontist? or prosthodontist? or nurse or nurses or pharmacist? or prescriber? or provider?) adj3 (belief or beliefs)).tw,kf. (2060)

51 ((doctor? or family clinician? or family practitioner? or general practitioner? or GP or GPs or medical practitioner? or medical specialist? or physician? or dentist? or endodontist? or exodontist? or maxillofacial surgeon? or maxillo-facial surgeon? or oral surgeon? or periodontist? or prosthodontist? or nurse or nurses or pharmacist? or prescriber? or provider?) adj3 (bias or biased or biases or biasing)).tw,kf. (701)

52 ((doctor? or family clinician? or family practitioner? or general practitioner? or GP or GPs or medical practitioner? or medical specialist? or physician? or dentist? or endodontist? or exodontist? or maxillofacial surgeon? or maxillo-facial surgeon? or oral surgeon? or periodontist? or prosthodontist? or nurse or nurses or pharmacist? or prescriber? or provider?) adj3 empath*).tw,kf. (745)

53 ((doctor? or family clinician? or family practitioner? or general practitioner? or GP or GPs or medical practitioner? or medical specialist? or physician? or dentist? or endodontist? or exodontist? or maxillofacial surgeon? or maxillo-facial surgeon? or oral surgeon? or periodontist? or prosthodontist? or nurse or nurses or pharmacist? or prescriber? or provider?) adj3 knowledge*).tw,kf. (11554)

54 ((doctor? or family clinician? or family practitioner? or general practitioner? or GP or GPs or medical practitioner? or medical specialist? or physician? or dentist? or endodontist? or exodontist? or maxillofacial surgeon? or maxillo-facial surgeon? or oral surgeon? or periodontist? or prosthodontist? or nurse or nurses or pharmacist? or prescriber? or provider?) adj3 opinion*).tw,kf. (3208)

55 ((doctor? or family clinician? or family practitioner? or general practitioner? or GP or GPs or medical practitioner? or medical specialist? or physician? or dentist? or endodontist? or exodontist? or maxillofacial surgeon? or maxillo-facial surgeon? or oral surgeon? or periodontist? or prosthodontist? or nurse or nurses or pharmacist? or prescriber? or provider?) adj3 perception?).tw,kf. (9377)

56 ((doctor? or family clinician? or family practitioner? or general practitioner? or GP or GPs or medical practitioner? or medical specialist? or physician? or dentist? or endodontist? or exodontist? or maxillofacial surgeon? or maxillo-facial surgeon? or oral surgeon? or periodontist? or prosthodontist? or nurse or nurses or pharmacist? or prescriber? or provider?) adj3 prejudic*).tw,kf. (89)

57 ((doctor? or family clinician? or family practitioner? or general practitioner? or GP or GPs or medical practitioner? or medical specialist? or physician? or dentist? or endodontist? or exodontist? or maxillofacial surgeon? or maxillo-facial surgeon? or oral surgeon? or periodontist? or prosthodontist? or nurse or nurses or pharmacist? or prescriber? or provider?) adj3 (value or values)).tw,kf. (3011)

58 clinical* practi#e*.tw,kf. (153384)

59 medical practice*.tw,kf. (19222)

60 prescriber? practi#e*.tw,kf. (24)

61 ((doctor? or family clinician? or family practitioner? or general practitioner? or GP or GPs or medical practitioner? or medical specialist? or physician? or dentist? or endodontist? or exodontist? or maxillofacial surgeon? or maxillo-facial surgeon? or oral surgeon? or periodontist? or prosthodontist? or nurse or nurses or pharmacist? or prescriber? or provider?) adj3 behav*).tw,kf. (7533)

62 (barrier? adj3 (care or healthcare or health care)).tw,kf. (6774)

63 exp Motivation/ (153146)

64 ((doctor? or family clinician? or family practitioner? or general practitioner? or GP or GPs or medical practitioner? or medical specialist? or physician? or dentist? or endodontist? or exodontist? or maxillofacial surgeon? or maxillo-facial surgeon? or oral surgeon? or periodontist? or prosthodontist? or nurse or nurses or pharmacist? or prescriber? or provider?) adj3 (motive? or motivat*)).tw,kf. (1817)

65 ((doctor? or family clinician? or family practitioner? or general practitioner? or GP or GPs or medical practitioner? or medical specialist? or physician? or dentist? or endodontist? or exodontist? or maxillofacial surgeon? or maxillo-facial surgeon? or oral surgeon? or periodontist? or prosthodontist? or nurse or nurses or pharmacist? or prescriber? or provider?) adj3 (aspire* or aspiring or aspiration*)).tw,kf. (244)

66 ((doctor? or family clinician? or family practitioner? or general practitioner? or GP or GPs or medical practitioner? or medical specialist? or physician? or dentist? or endodontist? or exodontist? or maxillofacial surgeon? or maxillo-facial surgeon? or oral surgeon? or periodontist? or prosthodontist? or nurse or nurses or pharmacist? or prescriber? or provider?) adj3 (inspire* or inspiring or inspiration*)).tw,kf. (212)

67 ((doctor? or family clinician? or family practitioner? or general practitioner? or GP or GPs or medical practitioner? or medical specialist? or physician? or dentist? or endodontist? or exodontist? or maxillofacial surgeon? or maxillo-facial surgeon? or oral surgeon? or periodontist? or prosthodontist? or nurse or nurses or pharmacist? or prescriber? or provider?) adj3 (incentiv* or disincentiv*)).tw,kf. (1538)

68 exp Professional-Patient Relations/ (133177)

69 (patient? adj (relations or relationship?)).tw,kf. (24179)

70 exp Professional Role/ (77458)

71 ((doctor? or family clinician? or family practitioner? or general practitioner? or GP or GPs or medical practitioner? or medical specialist? or physician? or dentist? or endodontist? or exodontist? or maxillofacial surgeon? or maxillo-facial surgeon? or oral surgeon? or periodontist? or prosthodontist? or nurse or nurses or pharmacist? or prescriber? or provider?) adj3 (role or roles)).tw,kf. (23607)

72 exp Social Responsibility/ (22470)

73 ((moral* or social*) adj2 responsib*).tw,kf. (2651)

74 accountab*.tw,kf. (15542)

75 Delivery of Health Care/ (78558)

76 ((care or healthcare or health care or patient care) adj2 (deliver* or system?)).tw,kf. (90970)

77 Practice Patterns, Physicians'/ (51683)

78 Practice Patterns, Dentists'/ (2033)

79 Practice Patterns, Nurses'/ (2065)

80 practice pattern?.tw,kf. (7130)

81 prescrib* pattern?.tw,kf. (2539)

82 Clinical Competence/ (81687)

83 (clinical* adj2 competen*).tw,kf. (3555)

84 ((doctor? or family clinician? or family practitioner? or general practitioner? or GP or GPs or medical practitioner? or medical specialist? or physician? or dentist? or endodontist? or exodontist? or maxillofacial surgeon? or maxillo-facial surgeon? or oral surgeon? or periodontist? or prosthodontist? or nurse or nurses or pharmacist? or prescriber?) adj2 competen*).tw,kf. (2777)

85 clinical* skill*.tw,kf. (5188)

86 exp Clinical Audit/ (21059)

87 ((clinical or dental or medical or nurs*) adj audit?).tw,kf. (3151)

88 ((clinical or therapeutic* or treatment) adj inertia?).tw,kf. (475)

89 or/34-88 [CLINICAL INERTIA TERMS] (1217774)

90 33 and 89 (3098)

91 exp Child/ not (exp Adult/ or Adolescent/) (756143)

92 exp Infant/ not (exp Adult/ or Adolescent/) (659866)

93 90 not (91 or 92) [CHILD-ONLY REMOVED] (3032)

94 exp Animals/ not (exp Animals/ and Humans/) (4438182)

95 93 not 94 [ANIMAL-ONLY REMOVED] (3015)

***************************
